# Immunisation with purified *Coxiella burnetii* phase I lipopolysaccharide confers partial protection in mice independently of co-administered adenovirus vectored vaccines

**DOI:** 10.1016/j.vaccine.2023.04.012

**Published:** 2023-05-05

**Authors:** Christina Dold, Henderson Zhu, Laura Silva-Reyes, Luke Blackwell, Aline Linder, Kevin Bewley, Kerry Godwin, Susan Fotheringham, Sue Charlton, Young Chan Kim, Andrew J. Pollard, Christine S. Rollier

**Affiliations:** a Oxford Vaccine Group, Department of Paediatrics, University of Oxford, and the National Institute for Health Research (NIHR) Oxford Biomedical Research Centre, Oxford, UK; b UKHSA Porton Down, Medical Interventions Group, Salisbury, Wiltshire, UK; c Section of Immunology, School of Biosciences, Faculty of Health and Medical Sciences, University of Surrey, Guildford, UK

**Keywords:** Adenoviral vector, *Coxiella burnetii*, Lipopolysaccharide, Q fever, Preclinical, Vaccine

## Abstract

Q fever is a highly infectious zoonosis caused by the Gram-negative bacterium *Coxiella burnetii.* The worldwide distribution of Q fever suggests a need for vaccines that are more efficacious, affordable, and does not induce severe adverse reactions in vaccine recipients with pre-existing immunity against Q fever. Potential Q fever vaccine antigens include lipopolysaccharide (LPS) and several *C. burnetii* surface proteins. Antibodies elicited by purified *C. burnetii* lipopolysaccharide (LPS) correlate with protection against Q fever, while antigens encoded by adenoviral vectored vaccines can induce cellular immune responses which aid clearing of intracellular pathogens. In the present study, the immunogenicity and the protection induced by adenoviral vectored constructs formulated with the addition of LPS were assessed. Multiple vaccine constructs encoding single or fusion antigens from *C. burnetii* were synthesised. The adenoviral vectored vaccine constructs alone elicited strong cellular immunity, but this response was not correlative with protection in mice. However, vaccination with LPS was significantly associated with lower weight loss post-bacterial challenge independent of co-administration with adenoviral vaccine constructs, supporting further vaccine development based on LPS.

## Highlights

• Adenoviral vectored vaccine constructs against Q fever were synthesised.
• Vaccine constructs elicited robust antigen-specific T cell immunity in mice.
• Comparable T cell immunity when co-administering several vaccine constructs.
• Formulating constructs with LPS or LPS alone gave protection against challenge.

## 1 Introduction

Q fever is a highly infectious, globally distributed zoonotic disease caused by the Gram-negative intracellular bacteria *Coxiella burnetii* [1]. While it has long been appreciated that many animal species can be infected with *C. burnetii*, aerosols and dairy products derived from infected livestock, including cattle, sheep, and goats, are the primary infectious agents of Q fever in humans [[2], [3]]. This risk is particularly high during parturition. The high infectivity of *C. burnetii* is reflected in its ability to infect humans putatively with as little as a single bacterium, hence is listed as a select agent by the CDC in the US [4].

Acute Q fever infection is characterised by flu-like and non-specific clinical features such as fever, while also displaying subclinical features including chills and headache [5]. After initial infection, around 5 % of patients become chronically infected. Chronic infection is commonly characterised by endocarditis, hepatitis, and chronic fatigue syndrome, which requires long-term antimicrobial therapy [[6], [7], [8]]. In addition to the challenges of treating chronic Q fever, relapses are common and can be fatal [[9], [10]]. Moreover, the low clinical suspicion of Q fever also contributes to the misuse of antimicrobial drugs and hence the selection of antimicrobial resistant *C. burnetii* strains [[11], [12]]. Therefore, humans and livestock at risk can benefit from immunisation with safe and protective vaccines against Q fever to reduce potential spread and the use of antimicrobial therapies.

Both inactivated whole cell vaccines and chemical extracts of *C. burnetii* surface antigens have been proposed as vaccine design approaches for human and veterinary use against Q fever. Inactivated whole cell vaccines progressed further into licensure, with one human inactivated whole cell vaccine (Q-Vax®) licensed in Australia, and a veterinary inactivated vaccine (Coxevac®) licensed in the EU. However, Q-Vax can induce severe adverse reactions in humans with pre-existing immunity to *C. burnetii* [[13], [14]]. Thus, pre-vaccination screening for existing immunity became an essential procedure prior to vaccination, although several severe local reactions have also been reported despite negative pre-vaccination testing results [[15], [16], [17]]. Efforts have been made to improve the safety profile of the whole cell vaccine by genetically attenuating the virulence of the *C. burnetii* Nine Mile phase I strain, which is the strain used in Q-Vax®, through the removal of the type IVB secretion system. The attenuated strain was tested in a guinea pig model, and a reduction in reactogenicity was observed [18]. Chemical extraction methods have also been intensively investigated with the aim of finding a safer alternative to the inactivated vaccine. Surface antigens purified using chloroform–methanol extraction and more recently detergent extraction elicited robust immune responses in animal models with reduced adverse reactions compared with inactivated Q fever vaccines [[16], [19]]. However, both inactivated vaccines and chemically extracted antigens are costly due to the requirement of biosafety level 3 (BSL-3) manufacturing facilities to produce the initial *C. burnetii* culture [20]. Inactivated vaccines require propagation of *C. burnetii* in embryonated hen eggs which may raises ethical and reproducibility concerns [21]. Several alternative approaches that do not require BSL-3 facilities have undergone preclinical evaluations. These approaches include generating peptide mimics of *C. burnetii* lipopolysaccharide (LPS), which demonstrated protection in mice [22], and using a mix of soluble proteins extracted from an avirulent *C. burnetii* Nine Mile phase II strain which allows for propagation and handling under biosafety level 2. This protein extract shown similar levels of protection compared with the whole cell vaccine in mice without inducing hypersensitivity [19]. Overall, while there are various vaccine approaches under development for Q fever, a more cost-effective and safer option would be better suited for use in low- and middle-income settings.

As understanding of the immune response to Q fever becomes increasingly refined, antibody responses to the smooth *C. burnetii* phase I LPS have been correlated with protection while response to the rough phase II LPS was not protective [[18], [22], [23], [24]]. Experimental evidence also implicates *C. burnetii* surface proteins as viable vaccine antigens [[15], [25]]. As an intracellular pathogen, *C. burnetii* infects, survives, and replicates within host cells. Hence, vaccines that elicit cellular immunity and humoral immune response may be more efficacious. Cellular immunity can be achieved by activating antigen-specific CD8+ T cells to enable the killing of *C. burnetii-*infected cells [26]. Taken together, the optimal vaccine formulation would favour including components that can induce both cellular and humoral immunity against *C. burnetii*.

The present work explores using replication-incompetent adenovirus vectors encoding Q fever protein antigens, formulated with or without phase I LPS as vaccine candidates against Q fever. Adenovirus vector vaccines have been extensively documented for their ability to induce strong CD8+ T cell responses while also inducing robust and long-lasting humoral responses to the encoded antigen [[27], [28], [29]]. The utility of the adenovirus vector vaccine platform has recently been recognised by the use of ChAdOx1 nCoV-19 (AZD1222) and Ad26.COV2-S (Jcovden) worldwide against SARS-CoV-2, which showed high efficacy, safety, and scalability under a global breakout setting [[28], [30], [31]]. Using both mouse immunogenicity and challenge models, we observed that adenoviral vaccine constructs elicited T cell responses in mice. No significant interference in antigen-specific T cell responses was observed when up to five adenovirus vectored constructs were administered concomitantly. In addition, T cell responses were maintained when adenovirus vectored constructs were co-administered with LPS. As Q fever is non-lethal in mice, analysing weight loss post-challenge can act as a surrogate of protection. However, the adenovirus vectored constructs did not induce a higher reduction in weight loss post-challenge when compared with LPS alone.

## 2 Results

### 2.1 Design of adenovirus vaccine constructs

Adenovirus vector vaccine constructs were created to encode an array of *C. burnetii* surface antigens identified from literature (Table 1). Individual transgenes described in Table 1 were inserted into E1 and E3 deleted human serotype 5 (HuAd5) adenoviral vectors, and verified by DNA sequencing with specific primers.

**Table 1:** Characteristics of the 14 transgenes inserted into individual HuAd5 vaccine vectors.

Transgene name	Entrez gene ID	Size (amino acid)	Location / function	Reference
Com1	1209823	252	Trans-membrane-associated protein, surface exposed.Catalyses intra-chain disulfide bond	[[32], [33], [34]]
Mip	1208515	230	Macrophage infectivity potentiator, outer membrane-associated protein	[[33], [34]]
SecB	1209429	161	Protein translocase, type 2 secretion system (T2SS)	[32]
P1	1208193	251	Outer membrane porin	[35]
OmpA	1209165	247	Outer membrane porin	[36]
GroEL	1209629	552	Chaperonin	[33]
OmpH	1208497	165	Outer membrane protein with one *trans*-membrane domain, molecular chaperone	[33]
YbgF	1207962	305	Periplasmic component of Tol system	[[33], [34]]
IcmG	1209537	244	Involved in Type 4 secretion system (T4SS)	[34]
IcmK	1209539	351	Involved in Type 4 secretion system (T4SS)	[34]
CirD	1209965	300	Substrate of the T4SS	[34]
CBU1157	1209059	233	Putative exported lipoprotein	[37]
CBU0091	1207961	195	OmpA-like, predicted to be situated on the outer membrane and be secreted	[38]
CBU1652	1209563	376	IcmX, T4SS	[39]

### 2.2 T cell responses are induced by immunization with adenoviral vectors, and are maintained when up to five constructs are administered concomitantly

**Fig. 1:**
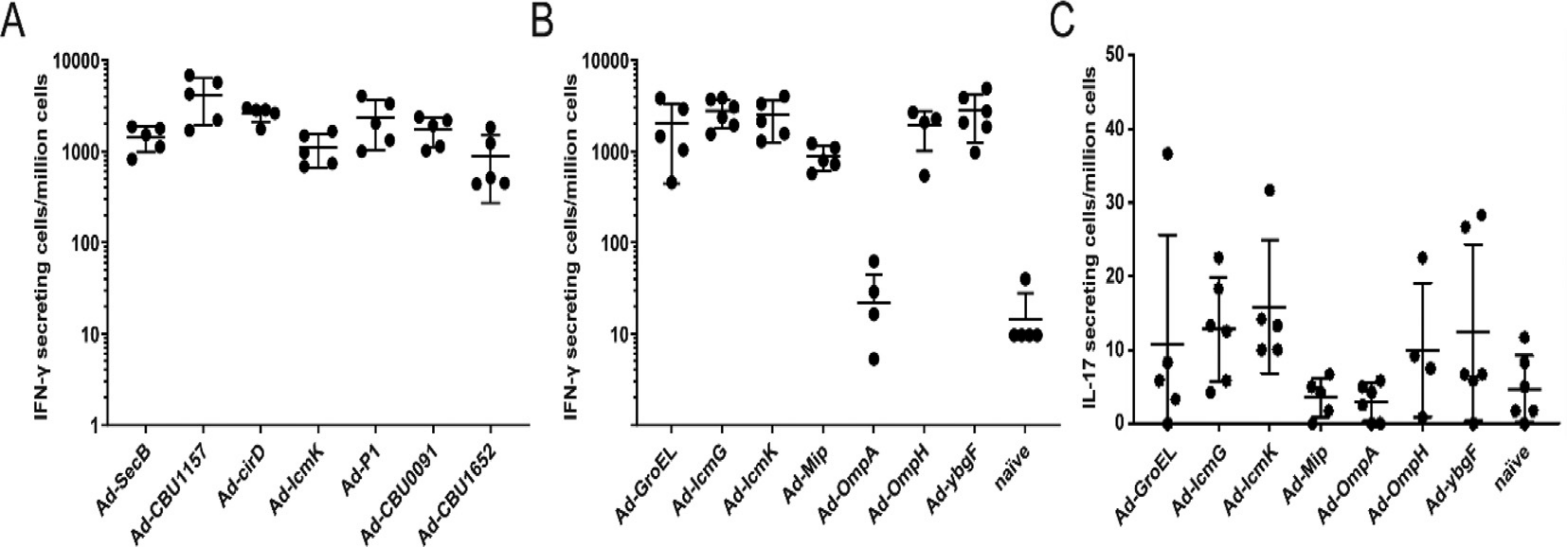
Antigen-specific IFN- γ and IL-17 producing cells elicited in mice immunised with adenoviral vaccine constructs expressing the selected Q fever antigens. (A) Quantification of IFN-γ producing cells two weeks post injection. Groups of mice were immunised at day 0 with the vaccines indicated in the X axis. Cellular responses (spots forming units/million) were enumerated in splenocytes at week 2. Each dot represents an individual mouse, and the horizontal bar indicates the mean and standard deviation of the group. (B) Quantification of IFN-γ producing cells two weeks post injection. Groups of mice were immunised at day 0 with the vaccines indicated in the X axis. Cellular responses (spots forming units/million) were enumerated in splenocytes at week 2. Controls mice were naïve. Each dot represents an individual mouse, and the horizontal bar indicates the mean and standard deviation of the group. (C) Quantification of IL-17 producing cells. The same group of mice as shown in panel (B) were immunised at day 0 with the vaccines indicated in the X axis. Cellular responses were enumerated in splenocytes at week 2. Controls mice were naïve mice. Each dot represents an individual mouse, and the horizontal bar indicates the mean and standard deviation of the group.

To assess the induction of a cellular response by a series of adenovirus vaccine constructs, groups of mice (n = 5) were immunised with one of fourteen different adenoviral vaccine candidates, each expressing a single antigen, at a dose of 1x10^9^ infectious units per mice. Antigen-specific interferon-γ (IFN- γ) secreting cells were quantified in splenocytes 2 weeks post immunisation (Fig. 1A; Fig. 1B). All constructs except for Ad-OmpA induced T cell responses ranging from a geometric mean of 455 (Ad-MIP) to 3601 (Ad-CBU1157) IFN-γ secreting cells per million splenocytes (spots forming units/million; S.F.U./million). Interleukin 17 (IL-17) responses were measured in splenocytes for selected Q fever antigens and the adenoviral vectors encoding IcmG (geometric mean 5.381) and IcmK (geometric mean 4.581) induced a low level of IL-17 response in all mice immunised (Fig. C).

**Fig. 2:**
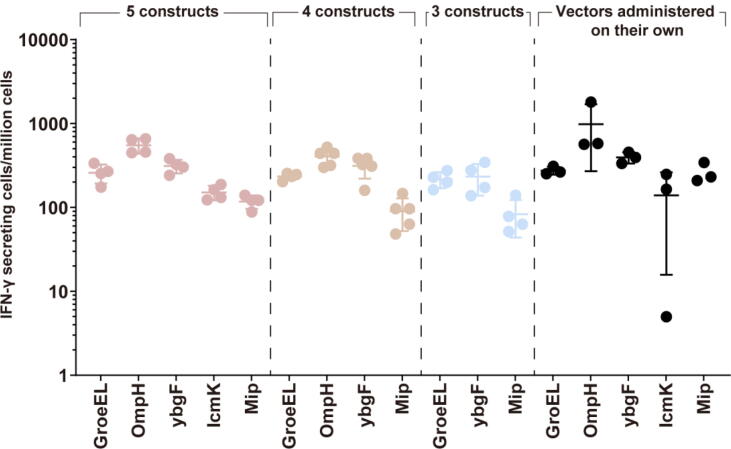
Antigen-specific Interferon-γ producing cells elicited in mice immunised with mixes of adenoviral vaccine constructs expressing selected Q fever antigens, as compared with the vaccines injected on their own. Groups of mice were immunised at day 0 with: A mix of 5 adenoviral (Ad) components (left panel, pink); A mix of 4 components: Ad-OmpH, Ad-YbgF, Ad-MIP or Ad-GroEL (second panel, orange); A mix of 3 components: Ad-YbgF, Ad-MIP or Ad-GroEL (third panel, purple); Adenoviral vaccines Ad-IcmK, Ad-OmpH, Ad-YbgF, Ad-MIP or Ad-GroEL administered on their own (right panel, black dots). Cellular responses were enumerated in splenocytes at week 2. The graphs depict the response to each individual antigen used. Each dot represents an individual mouse, and the horizontal bar indicates the geometric mean and standard deviation of the group. (For interpretation of the references to colour in this figure legend, the reader is referred to the web version of this article.)

To assess the effects of co-immunisation of multiple vaccine constructs, groups of mice received a mix of either three, four or five vaccine constructs at a dose of 2 × 10^8^ infectious units per vaccine construct per mice, while another group of mice received a single vaccine construct at 2x10^8^ infectious units per mice (Fig. 2). Adenoviral constructs that elicited higher antigen-specific IFN-γ producing cells from one of the single vaccine construct assessments (Fig. 1B) were used. Statistical significances were calculated between the adenoviral vectored vaccine constructs encoding the same antigen with Kruskal-Wallis test followed by Dunn's multiple comparisons test. The results show that antigen-specific IFN-γ responses from the co-immunisation of multiple vaccines were not significantly different compared with immunisation with a single vaccine construct (Adj. P > 0.9999). The levels of IFN-γ response for each construct was maintained even when given as part of three, four or five co-immunisation regimens. Ad-MIP and Ad-OmpH consistently induced the lowest and highest IFN-γ responses, respectively, while Ad-GroEL, Ad-IcmK and Ad-YbgF induced similar IFN-γ levels regardless of co-immunisation or administered alone.

### 2.3 Identification of three antigens that impact on Q fever infection

**Fig. 3:**
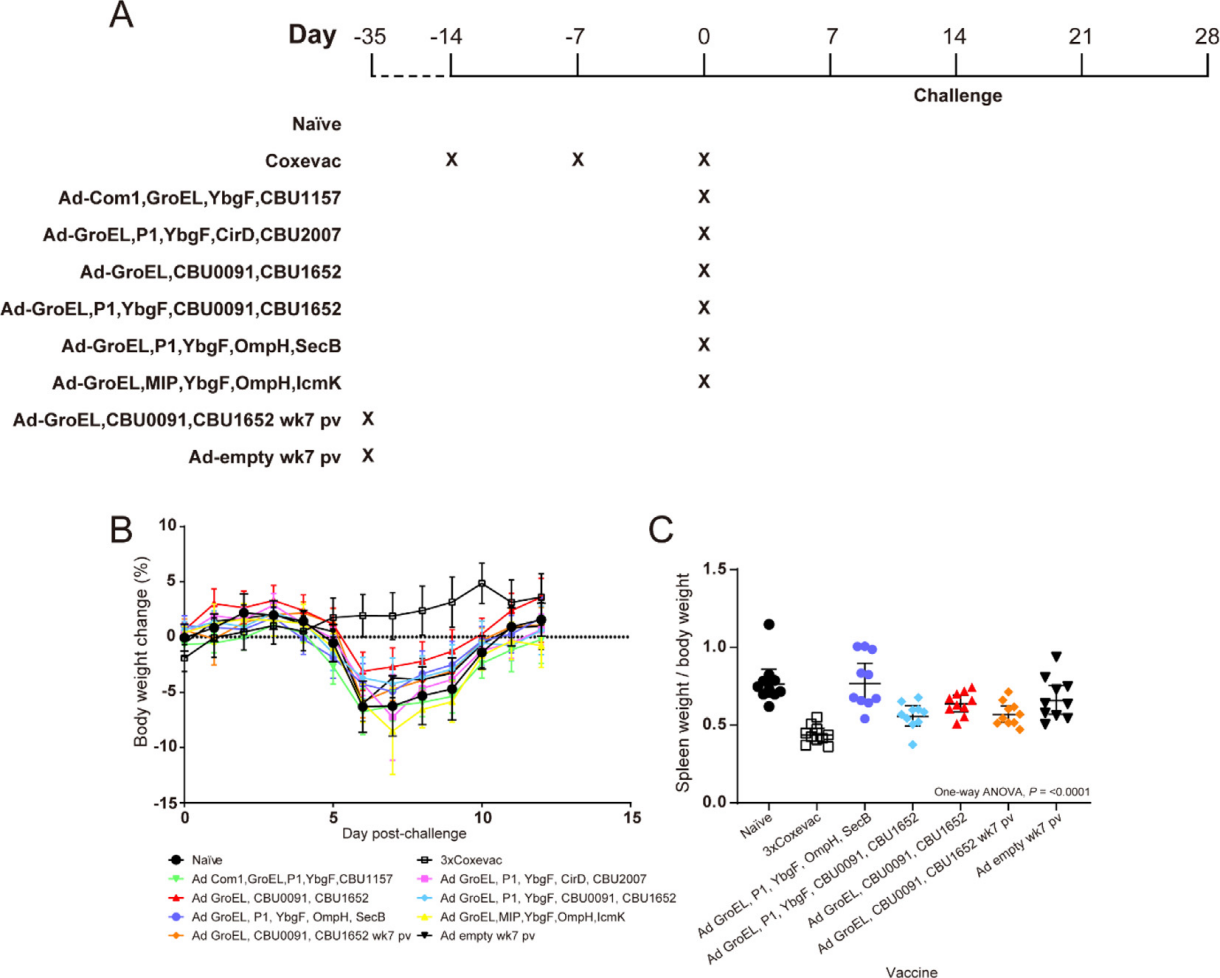
Mice challenged after immunisation with adenoviral vaccine constructs. (A) Mice (n = 10) were immunized with the vaccines at time points labelled by X, then challenged with aerosolised *C. burnetii.* (B) Body weight change post-challenge, dotted line indicates zero on the y-axis. Each point indicates the mean per group and error bars indicate 95 % CI. (C) Spleen weight/body weight ratio. Horizontal bars indicate geometric means and 95 % CI. Week 7 post-vaccination is abbreviated as wk7 pv. Significance is generated using one-way ANOVA.

We then used an *in vivo* challenge model to assess the protection conferred by the adenoviral vaccine constructs. Mice were immunised and challenged two weeks post-immunisation with several adenoviral vaccine constructs (Supplementary Table 1). Weight changes post-challenge in mice receiving adenoviral vaccine constructs was compared with positive control mice immunised with an inactivated Q fever vaccine, Coxevac®, in a prime-boost-boost regimen. An adenoviral vector that does not encode a transgene (Ad-empty) was used as a negative control alongside the naïve group (Fig. 3; Supplementary Table 1). Several combinations of adenoviral vectored constructs were assessed in the aim to assess the protection of these constructs.

In comparison with the naïve control, immunization with the positive control Coxevac induced the highest reduction in weight loss at DPI = 7 and DPI = 8 post-challenge, followed by a mix of Ad-GroEL, Ad-CBU0091, and Ad-CBU1652, then by a mix of Ad GroEL, Ad-P1, Ad-YbgF, Ad-CBU0091, and Ad-CBU1652, which was then followed by a mix of Ad-GroEL, Ad-CBU0091, and Ad-CBU1652 (challenged 7-weeks post immunisation). The trend was consistent when the spleen weight/body weight ratio was examined. Mouse receiving Coxevac (*P* = <0.0001), or a mix of Ad-GroEL, Ad-P1, Ad-YbgF, Ad-CBU0091, and Ad-CBU1652 (*P* = 0.0016), or a mix of Ad-GroEL, Ad-CBU0091, and Ad-CBU1652 (7-weeks post-vaccination; *P* = 0.0031) showed ratios significantly lower than the naïve control when compared using one-way ANOVA followed by Tukey’s multiple comparison test (Fig. 3B). Two groups of mice that were 7-weeks post-vaccination were challenged to assess for potential persistence of protection and to include a negative group that received an adenoviral vector encoding no antigen. Mouse administered a mix of Ad-GroEL, Ad-P1, Ad-YbgF, Ad-OmpH, and Ad-SecB did not demonstrate a significantly lower spleen weight/body weight ratio compared with the naïve control. Overall, we observed that adenoviral vectored vaccine formulations incorporating Ad-CBU0091 and Ad-CBU1652 resulted in more reduction in weight loss post-challenge compared with other adenoviral formulations.

While the Coxevac group retained the highest body mass post-challenge, concomitant immunisation of Ad-GroEL, Ad-CBU0091, and Ad-CBU1652 was able to maintain the highest body mass within the adenovirus vector groups, followed by a mix of Ad-GroEL, Ad-P1, Ad-YbgF, Ad-CBU0091, and Ad-CBU1652. The mice immunized with a mix of Ad-Com1, Ad-GroEL, Ad-P1, Ad-YbgF, and Ad-CBU1157 had the lowest body mass post-challenge. The ability for the concomitant immunisation of Ad-GroEL, Ad-CBU0091, and Ad-CBU1652 to prevent weight loss when challenged 7-weeks post-immunisation was decreased, however a reduction in weight loss was observed as compared with the other adenoviral vaccine constructs.

### 2.4 Development of adenoviral vectored vaccine against Q fever encoding fusion antigens composed of GroEL, CBU0091, and CBU1652

**Fig. 4:**
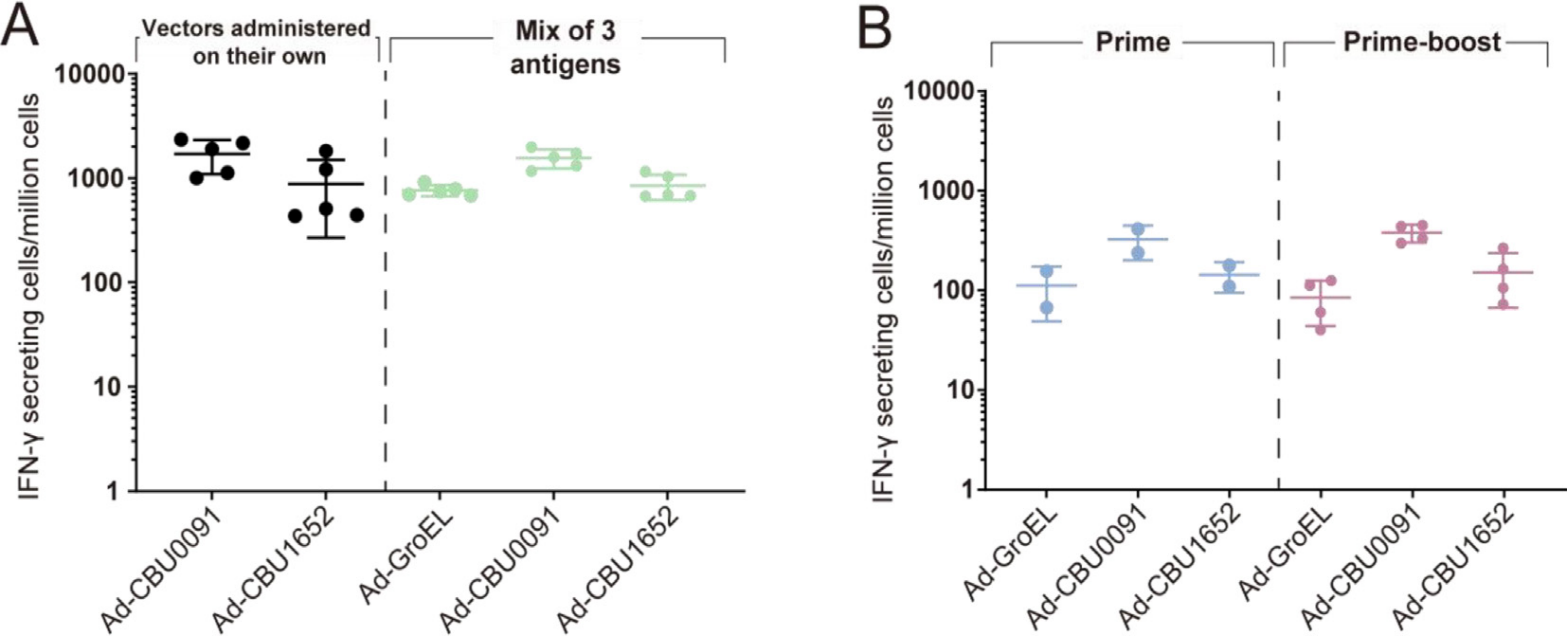
Antigen-specific Interferon-γ producing cells elicited in mice immunised with adenoviral vectored vaccine constructs administered under different regimens. (A) Comparing vaccine constructs administered on their own or concomitantly. Groups of mice (n = 5) were immunised at day 0 with Ad-CBU0091 or Ad-CBU1652 on their own (left panel); Concomitant administration of a mix of Ad-CBU0091 + Ad-CBU1652 + Ad-GroEL (right panel). Cellular responses were enumerated in splenocytes at week 2. Each dot represents an individual mouse, and the horizontal bars indicate the mean and the standard deviation. (B) Comparing vaccine constructs administered using a single-dose (prime) or a prime-boost regimen. Groups of mice were immunised concomitantly at day 0 with Ad-GroEL + Ad-CBU0091 + Ad-CBU1652 (left panel); Ad-GroEL + Ad-CBU0091 + Ad-CBU1652 administered at day 0 and week 8 (right panel). Cellular responses were enumerated in splenocytes at week 10. The graphs depict the response to each individual antigen used. Each dot represents an individual mouse, and the horizontal bars indicate the mean and the standard deviation.

The observation that the group of mice receiving a mix of Ad-GroEL, Ad-CBU0091, and Ad-CBU1652 vaccination resulted in the least reduction in weight post-challenge prompted us to explore further this combination of adenoviral vaccines. No significant difference in the T cell responses was observed when the antigens were administered alone or concomitantly (Fig. 4A, Kruskal-Wallis with Dunn’s multiple comparison test, adjusted *P* values > 0.9999).

To explore if *T*-cell responses can be increased by a boost dose of these vaccine constructs, concomitant administration of Ad-GroEL, Ad-CBU0091, and Ad-CBU1652 was performed either once (week 0) or as a potential 2 dose regimen (week 0 and week 8). The homologous boost dose given 8 weeks after the prime did not significantly enhance the T cell response (Fig. 4B; Kruskal-Wallis with Dunn’s multiple comparison test, adjusted *P* values > 0.9999).

**Fig. 5:**
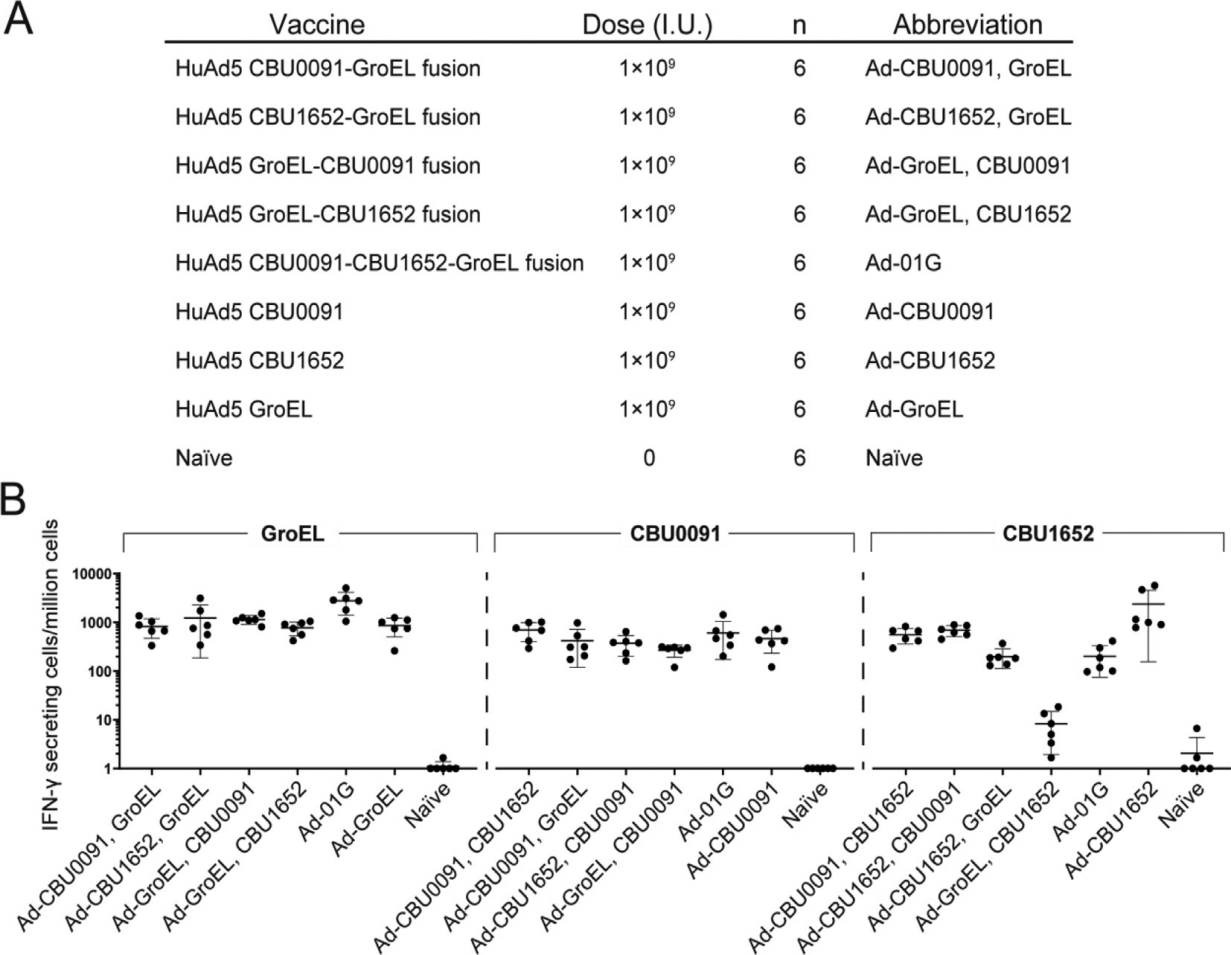
Antigen-specific Interferon-γ producing cells elicited by adenoviral vaccine constructs encoding fusion antigens. (A) Experimental design of the fusion vaccine constructs assessment. Dose is indicated by infectious units and number of mice are indicated by n. (B) Groups of mice (n = 6) were immunised at day 0 with adenoviral vectored vaccine constructs encoding double fusion antigens or a triple fusion antigen. Cellular responses were enumerated in splenocytes at week 2. Each dot represents an individual mouse, and the horizontal bars indicate the mean and the standard deviation. GroEL-specific responses are shown in left panel, responses specific to CBU0091 are shown in middle panel, and responses specific to CBU1652 are shown in the right panel.

Fusion constructs comprising two or three of these antigens were produced and assessed for T cell immunogenicity (Fig. 5). Triple fusion construct expressing CBU0091, CBU1652 and GroEL (Ad-01G) and double fusion constructs elicited similar levels of antigen-specific IFN-γ secretion, with the exception of the HuAd5 GroEL-CBU1652, which elicited a weaker response. The absence of immune interference with four out of the five fusion vaccine constructs may reflect that each antigen was being produced and presented without perturbation to their immunological properties for these. Ad-01G fusion demonstrated immunogenicity against all three antigens while being composed of only one adenoviral vector component, hence was further assessed with the addition of LPS.

### 2.5 LPS is the main driver of vaccine-induced reduction of Q fever infection

**Fig. 6:**
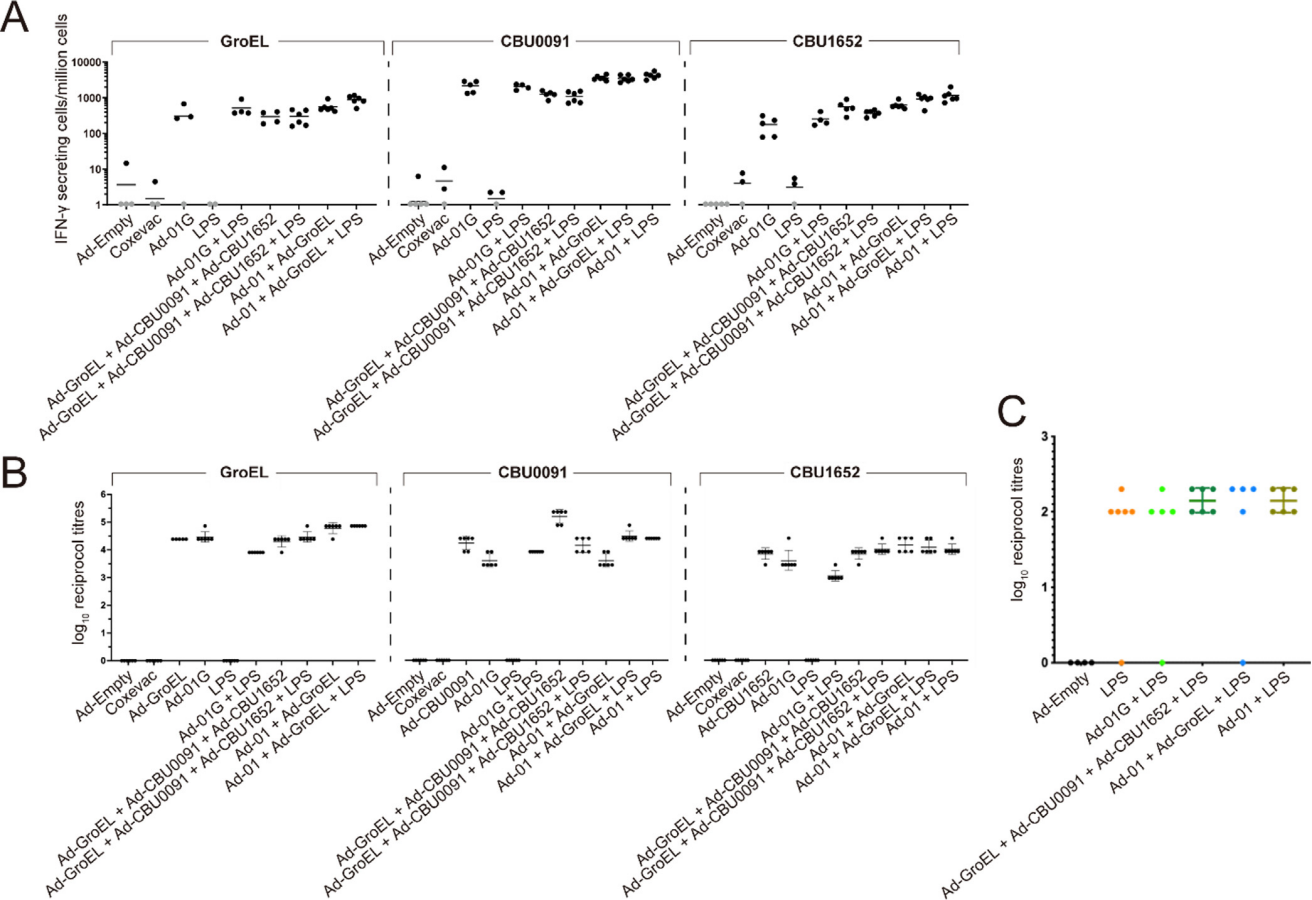
Humoral and cellular responses in mice immunised with Ad-01G triple fusion or mix of Ad-CBU0091/Ad-CBU1652/Ad-GroEL with or without *C. burnetii* phase I LPS. (A) Antigen-specific Interferon-γ producing cells. Mice with no IFN-γ secreting cell/million cell are highlighted in grey. Each dot represents an individual mouse, and the horizontal bars indicate the mean. (B) Humoral responses to the protein antigens in mice immunised with Ad-01G triple fusion or mix of Ad-CBU0091/Ad-CBU1652/Ad-GroEL with or without *C. burnetii* phase I LPS. The graph depicts the LPS antibody titres for each vaccine formulation. Each dot represents an individual mouse, and the horizontal bar indicates the geometric mean and standard deviation. (C) Anti-LPS IgG titres post-immunisation with adenoviral vaccine constructs formulated with *C. burnetii* phase I LPS. Each dot represents an individual mouse, and the horizontal bar indicates the geometric mean.

In spite of the humoral response to LPS being extensively documented as a correlate of protection against Q fever [[22], [23]], LPS are T cell independent antigen and do not elicit cellular immunity [40]. The T cell-stimulating properties of adenoviral vaccines were combined with antibody-inducing LPS to elicit both cellular and humoral immunity by preparing several vaccine candidates composed of the selected fusion adenoviral vaccine constructs formulated with or without LPS (Fig. 6A). The presence of LPS did not impact on the adenovirus-driven T cell responses, as observed by the similar level of IFN-γ secreting cells post vaccination of Ad-01G fusion, Ad-CBU0091 + Ad-CBU1652 + Ad-GroEL mix, and double fusion encoding CBU0091 and CBU1652 (Ad-01 fusion) + Ad-GroEL mix with or without LPS (Fig. 6A).

Similar levels of anti-CBU0091, anti-CBU1652, and anti-GroEL IgG responses were elicited by either administrating as a single construct, fusions or a mixed formulation (Fig. 6B), except for the triple fusion. Immunisation with the fusion Ad-01G vaccine induced reduction in IgG titres for all three antigens when compared with the constructs administered alone or as a mix. The addition of LPS to the adenoviral constructs also did not decrease the Ad-induced antibody responses. Finally, the anti-LPS IgG titres were measured using an anti-LPS enzyme-linked immunosorbent assay (ELISA). LPS alone and all adenoviral vaccines mixed with LPS elicited similar levels of anti-LPS IgG titres (Fig. 6C).

**Fig. 7:**
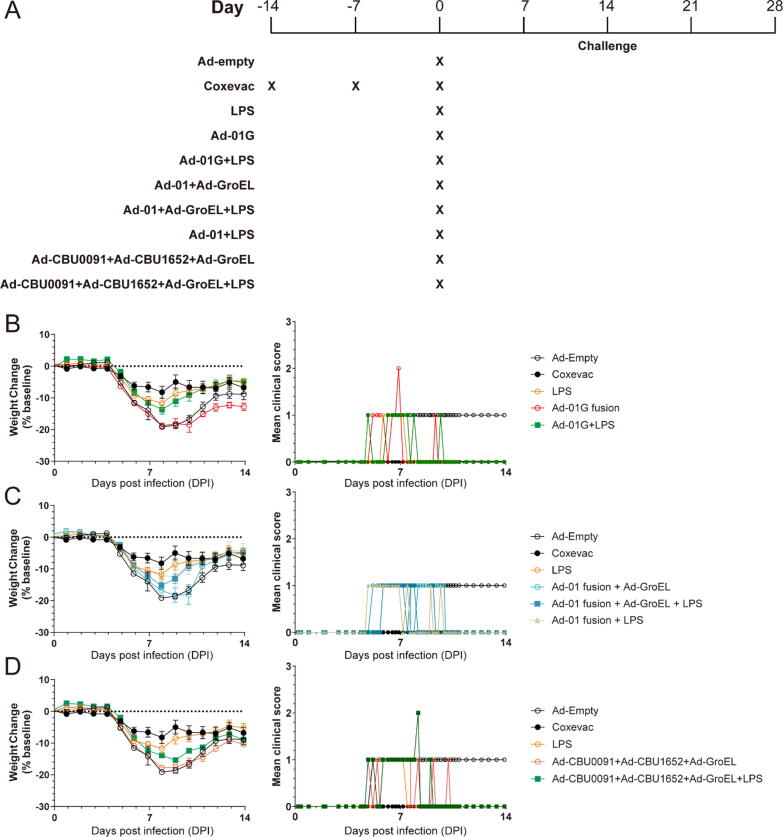
Mice challenged after immunisation with adenoviral vaccine constructs formulated with or without *C. burnetii* phase I LPS. (A) Timeline and vaccines administered. Mice (n = 10) were immunized with the vaccines at time points labelled by X, then challenged with an aerosol containing *C. burnetii.* Weight changes and mean clinical score following challenge compared with Ad-Empty and Coxevac after immunisation with (B) Ad-01G triple fusion with or without LPS; (C) Ad-01 double fusion without without LPS; (D) Concomitant immunisation with Ad-CBU0091, Ad-CBU1652, and Ad-GroEL, with or without LPS. Each dot indicates the mean of weight change of the group, horizontal bars show the standard error. Clinical scores were calculated by the sum of health status observations, in which healthy = 0, ruffled fur = 1, dehydration = 1, arched back = 2, eyes shut = 2, wasp waist = 2, immobile = 9, dead = 10.

A challenge experiment was performed to assess the adenoviral vectored vaccine constructs with the addition of LPS. Weight changes post-challenge was examined in mice receiving different vaccine formulations (Fig. 7A). Coxevac was used as a positive control, while an empty adenovirus vector was used as a negative control (Ad-Empty). The Coxevac group had the least weight loss post-challenge, followed by Ad-01G fusion + LPS, LPS, Ad-01 fusion + LPS, Ad-01 fusion + Ad-GroEL + LPS, Ad-GroEL + Ad-CBU0091 + Ad-CBU1652 + LPS, Ad-empty, Ad-GroEL + Ad-CBU0091 + Ad-CBU1652, and Ad-01G fusion (Fig. 7; Supplementary Table 2).

Administration of adenoviral vector constructs did not result in significant reduction in weight loss at days post infection (DPI) = 7 and 8 compared when with the Ad-empty negative control, this includes Ad-01G, Ad-01 fusion + Ad-GroEL, and Ad-CBU0091 + Ad-CBU1652 + Ad-GroEL. However, administering LPS together with Ad-01 fusion resulted in significant reduction in weight loss compared with the Ad-empty group at DPI = 8. However, co-administration of LPS with Ad-01G fusion, Ad-01 fusion + Ad-GroEL, and a mix of Ad- GroEL + Ad-CBU0091 + Ad-CBU1652 did not show significant reductions in weight loss post-challenge compared with the Ad-empty group at DPI = 8. Administering LPS alone induced a significantly reduced weight loss at DPI = 7 and 8 compared with the Ad-empty. Clinical scores were similar between the groups, with two mice receiving adenoviral vectored constructs alone reaching a score of three before quickly recovering. Altogether, these results suggest that while mixing adenoviruses and LPS does not impact on the immunogenicity of each component, the LPS-induced immune response has the most pronounced impact on reducing Q fever infection.

## 3 Discussion

The potential of adenovirus vectored vaccine constructs, formulated with *C. burnetii* LPS, was assessed in the present study with the aim of inducing both cellular and humoral immune responses to provide protection against Q fever infection. Our results showed that adenoviral vectored vaccine constructs encoding several Q fever antigens induced strong T cell responses in mice after a single injection. Administering these vaccine constructs concomitantly also produced T cell responses equivalent to the responses when the constructs were used alone, as no significant immune interference was observed. The comparison of the antigen-specific T cell responses elicited by either single or multiple vaccine constructs can potentially be made more complete by additional negative controls, including the addition of a control group receiving Ad-empty. Using the challenge model readouts, we observed that adenoviral vaccine constructs alone did not induce protection against virulent Q fever challenge in mice, and moreover, the addition of these constructs to purified LPS did not increase the protection compared with LPS alone.

Antigen-specific T cell responses have been described which correlate with protection against a range of intracellular bacteria and viruses, including *Listeria monocytogenes*, *Salmonella* Typhimurium, *Mycobacterium tuberculosis*, SARS-CoV-2, and Zika virus [[41], [42], [43]]. Our results showed that the adenoviral vector constructs encoding either a single or multiple fused *C. burnetii* surface antigens were immunogenic as they induced strong T cell responses. While this is in line with the ability of the adenovirus vectored vaccine platform to elicit cellular immunity [44], antigen-specific T cell responses did not correlate with weight loss reduction post-challenge. In addition, quantification of CD8+ T cell populations may be of interest as CD8+ T cells were shown to have an important role in protecting mice against *C. burnetii* infection [45].

Previous clinical studies and experimental evidence implicates bystander interference in the concomitant administration of vaccines in humans [[46], [47]]. This phenomenon is most likely due to the competition of the limited resources in lymph nodes, including cytokines and cytokine inhibitors by separate immune responses. However, the present study confirmed that the concomitant immunisation of up to five adenoviral vaccine constructs did not induce bystander interference in T cell responses, reflected by the comparable levels of antigen-specific T cell responses when the adenovirus constructs were administered on its own or administered concomitantly. This is reminiscent of previous studies involving adenoviral vectored vaccines against human immunodeficiency virus (HIV) and hepatitis C virus (HCV), in which T cell responses were shown to be elicited against multiple antigens where a mix of vectors encoding different antigens were co-administered [[48], [49]]. This flexibility for introducing multiple constructs in a single immunisation may in turn consolidate the potential for developing multi-valent vaccines using adenoviral vector platforms. The feasibility of the adenoviral constructs to be administered as a prime-boost regimen was also examined. We show that the T cell response post-boost was comparable to the response after priming. This may be caused by the neutralising effect of the anti-adenovirus immune response induced after the first immunisation in mice, which limited the expression of the encoded antigen and subsequently reduced T cell activation [[50], [51]]. To evade this, heterologous prime-boost immunisations, such as using modified vaccinia Ankara virus (MVA) encoding *C. burnetii* surface antigens, may be used [29]. In humans, recent studies demonstrated that adenoviral vectored vaccines can boost humoral response using a homologous prime-boost regimen when immunising against SARS-CoV-2 [[28], [52]].

Our study confirms that administering *C. burnetii* phase I LPS confers protection against *C. burnetii* challenge in mice. In addition, we observed that the addition of LPS to the adenoviral vectored vaccine formulations did not alter the humoral response of the adenoviral vectored vaccines. Derivatives of LPS, including detoxified lipid A, have been described as adjuvants to enhance IFN-γ production [53]. However, no similar adjuvating effect is reflected from formulating adenoviral vector constructs with LPS, despite LPS IgG ELISA readouts reflecting that the LPS was immunogenic by successfully inducing humoral responses. LPS is a T cell-independent antigen, which on its own cannot recruit CD4+ T cells and the subsequent affinity maturation [54]. However, T cell-independent sugars can be modified to induce CD4+ T cell recruitment by conjugating to an immunogenic carrier protein. Five carrier proteins have been used in licensed glycoconjugate vaccines, including *Haemophilus* protein D, tetanus toxoid, diphtheria toxoid, cross-reactive material 197 (CRM197, a non-toxic mutant form of diphtheria toxoid), and serogroup B meningococcus outer membrane protein complex (OMPC) [55]. Conjugating sugar-based antigens to a carrier protein enables T cell help in inducing B cell affinity maturation, clonal expansion, and memory responses [56]. Capsular polysaccharides (CPS)-based glycoconjugates have been used in several licensed vaccines against *Neisseria meningitidis, Haemophilus influenzae* type b, and *Streptococcus pneumoniae* [57]. To date, no LPS conjugate vaccine has been licensed due to several technical hurdles, mainly associated with the toxicity of the lipid A moiety of LPS, and with the low extraction yield of LPS from culture [[58], [59]]. However, recent advances in LPS harvesting and detoxification have shown to support LPS-based glycoconjugate production at larger scales [60]. Additionally, advances in synthetic glycology permits the synthesis of LPS mimics to be made, which avoids the requirement of biosafety level 3 facilities [61]. While most sugars can be synthesised, sugars specific to *C. burnetii* including virenose (6-deoxy-3-C-methylgulose) and dihydrohydroxystreptose (3-C-(hydroxymethyl)lyxose) within the *O*-specific polysaccharide of *C. burnetii* LPS have not been successfully synthesised to date [62]. Taken together, our result suggests that it is sensible to consolidate the utility of *C. burnetii* LPS as a vaccine antigen by conjugation with the aim to create the next generation of Q fever vaccines that is highly protective.

## 4 Materials and methods

### 4.1 Viral vector vaccines

Various *C. burnetii* antigens (Table 1) were inserted into the adenovirus human serotype 5 (AdHu5). The process of inserting antigens into the various vector platforms has been described previously [63]. Each antigen was inserted into a separate AdHu5 vector, and a selection were inserted together as a fusion polypeptide, facilitated by a flexible linker (ADLPSLAADFVE) into the same expression cassette in the vector.

For immunisations, regardless of the number of different adenovirus vaccines that were mixed for a single immunisation, a concentration of 1x10^9^ viral particles of infective adenoviral vector units were prepared in Dulbecco’s PBS.

### 4.2 LPS extraction

LPS was extracted using a modifed hot phenol-water method [[64], [65]]. Inactivated bacteria (Coxevac®; CEVA) was lysed using ultrasonication for 15 min followed by treatment with proteinase K (100 µg/ml; Roche) at 65 °C for one hour. The solution was then treated with RNAse (40 µg/ml; Roche), and DNAse (20 µg/ml; Roche), 20 % MgSO_4_ (1 µl/ml; Sigma-Aldrich), and chloroform (4 µl/ml; Sigma-Aldrich) at 37 °C overnight. Both the bacterial lysate solution and phenol was then subsequently heated to 70 °C and mixed by vigorous shaking. The mix was then rapidly cooled and centrifuged to separate the aqueous and the organic phases. The aqueous phase was extracted, and residual phenol removed by dialysis against PBS (3.5 K MWCO; Thermo Scientific). The purified solution was freeze-dried and stored at 4 °C.

### 4.3 Mouse immunisations

All procedures were performed in accordance with the terms of the U.K. Home Office Animals Act Project License and the UKHSA and University of Oxford Animal Care and Ethical Review Committee.

Six- to eight-week-old female C57BL/6 (H-2^b^) or A/J inbred mice (Envigo Laboratories) were anaesthetised prior to immunisation with Isoflo (Abbot Animal Health). Immunisations were administered intramuscularly with the adenoviral vaccine divided equally into each musculus tiabilis (25–50 µl per limb). Coxevac (supplied at 50 µg/ml) was diluted to 10 µg/ml in PBS, and 100 µl was administered intramuscularly per mouse. LPS extracted from inactivated *C. burentii* using a modified hot phenol-water method was diluted to 100 µg/ml and 100 µl was administered per mouse. Cardiac puncture was undertaken under anaesthesia and two weeks post immunisation and immune responses were assayed as described below.

### 4.4 Mouse challenge studies

The challenge agent *Coxiella burnetii* (Nine Mile strain) was grown in acidified citrate cysteine medium. Following growth, bacteria was pelleted and suspended in buffer for the challenge. The challenge is administered by aerosol at a concentration of approximately 1x10^9^ copies/ml. Aerosol delivery was performed through head-only exposure using a small particle aerosol delivery system (Biaera) at 65 % RH for 10 mins. Aerosols were generated in a 6 jet Collison nubuliser running at 15 L/min. Air samples were taken from the flow using an AGI-39 impinger into PBS. The positive control consisted of mice immunised three times with a veterinary Q fever vaccine, Coxevac® (CEVA). Two negative control groups were used, consisting of naïve mice, and mice immunised with an empty adenovirus vector.

### 4.5 T cell responses

T cell responses were quantified by using a mouse FluoroSpot kit (Mabtech, FSP-4144-10). Briefly, spleens were harvested from mice, and each placed in 5 ml autoMACS® Running Buffer in a gentleMACS C-Tube (Miltenyi Biotec). Cells were mechanically separated using the spleen program on the gentleMACs™ Dissociator (Miltenyi Biotec) and red blood cells (RBC) were lysed with RBC lysis buffer (Qiagen). The lysis reaction was quenched using autoMACs Rinsing solution (Miltenyi Biotec) and cells were filtered using 40 µm strainers (Greiner Bio-One, U.K.). The final cell preparation was resuspended in autoMACs Rinsing solution and viability was quantified using the Muse cell counter (Luminex). The splenocytes concentration was then adjusted to 4x10^6^ cells/ml with DMEM + 10 %FBS and 50 μl/well was added to low fluorescence elispot plates coated with anti-IFN-γ and anti-IL17A monoclonal antibodies at 15 μg/ml and 10 μg/ml, respectively. Peptide pools consisting of 15-mer sequences with 11-amino acid overlaps were created for each antigen and diluted at 6 μg/ml. Cells were then stimulated for 18–20 h with 50 μl of these peptide pools (3 μg/ml final concentration). The following day, detection of spots was carried out according to the manufacturer’s instructions (Mabtech) and analysed with AID ELISpot software 8.0 (Autoimmun Diagnostika).

### 4.6 Enzyme-linked immunosorbent assay (ELISA)

To perform CBU0091, CBU1652, and GroEL ELISAs, 0.25 μg of CBU0091, 0.25 μg of CBU1652, and 0.5 μg of GroEL were coated onto each well of a 96-well plate (Invitrogen) at final concentrations of 2.5 μg/ml, 2.5 μg/ml, and 5 μg/ml, respectively, and incubated at 4 °C overnight. The plate was blocked by 1 % BSA (Sigma-Aldrich) in PBS at 37 °C for two hours followed by six washes of 0.05 % Tween 20 (Sigma-Aldrich) in PBS (PBS-T) and the addition of mice serum with serial dilutions of 1/300, 1/900, 1/2700, 1/8100, 1/24300, 1/72900, and 1/218700 in 1 % BSA in PBS for 2 h at 20 °C. Following another six washes with PBS-T, peroxidase AffiniPure goat anti-mouse IgG (Jackson ImmunoResearch) was added and incubated for two hours at 20 °C. Following further six washes with PBS-T, 3,3′,5,5′-Tetramethylbenzidine (TMB; Sigma-Aldrich) was added and incubated for 5–15 min until no colour change is apparent and the reaction was stopped using 0.16 M sulphuric acid. Each plate was read at 450 nm and 630 nm and analysed using BioTek Gen 5 software (Agilent). Serum antibody reciprocal titres were calculated by an absorbance value three standard deviations greater than the average of OD 450 nm of the control.

To perform LPS ELISA, 0.25 μg LPS was coated onto each well of a 96-well plate (Invitrogen) at a final concentration of 5 μg/ml and incubated at 4 °C overnight. The plate was blocked by 1 % BSA (Sigma-Aldrich) in PBS at 37 °C for two hours followed by six washes of 0.05 % Tween 20 (Sigma-Aldrich) in PBS (PBS-T) and the addition of mice serum with serial dilutions of 1/100, 1/200, 1/400, and 1/800 in 1 % BSA in PBS for 2 h at 20 °C. Following another six washes with PBS-T, peroxidase AffiniPure goat anti-mouse IgG (Jackson ImmunoResearch) was added and incubated for two hours at 20 °C. Following further six washes with PBS-T, 3,3′,5,5′-Tetramethylbenzidine (TMB; Sigma-Aldrich) was added and incubated for 30–50 min until no colour change is apparent and the reaction was stopped using 0.16 M sulphuric acid. Each plate was read at 450 nm and 630 nm and analysed using BioTek Gen 5 software (Agilent). Serum antibody reciprocal titres were calculated by an absorbance value three standard deviations greater than the average of OD 450 nm of the control.

### 4.7 Statistics

Significance levels for weight changes were calculated with one-tailed one-way ANOVA for DPI = 7 (*P* < 0.0001) and DPI = 8 (*P* < 0.0001) followed by two-tailed Tukey’s multiple comparisons test. Significance levels for the spleen weight/body weight ratio were calculated using one-way ANOVA. For the comparison of antigen-specific IFN-γ producing cells elicited in mice, significance levels were calculated using Kruskal-Wallis test followed by Dunn’s multiple comparisons test if the Kruskal-Wallis test was rejected (*P* < 0.05).

## Contributions

C.S.R, and A.J.P conceived the project and obtained funding. C.D and C.S.R designed the experiments. C.D designed and constructed the vaccine constructs. C.D, L.B, A.L and L.S.R performed mice immunisations and immunogenicity assessments. K.B, K.G., S.F, and S.C. planned and performed the challenge experiments. H.Z and Y.C.K performed and analysed ELISA for humoral immunogenicity assessments. K.B, H.Z and Y.C.K extracted and characterised LPS. Y.C.K and H.Z optimised, performed, and analysed LPS ELISA. H.Z drafted the manuscript. Y.C.K, K.B, C.D, L.S.R, C.S.R and A.J.P reviewed and edited the manuscript. C.D., Y.C.K, A.J.P and C.S.R supervised the work.

## Funding

This research was funded in whole or in part by 10.13039/501100006041Innovate UK Vaccines for global development grant (project reference 971619). For the purpose of Open Access, the author has applied a CC BY public copyright licence to any Author Accepted Manuscript (AAM) version arising from this submission. Y.C.K is supported by the 10.13039/501100013373NIHR Oxford Biomedical Research Centre Small Grant and the 10.13039/100010269Wellcome Trust Grant (224117/Z/21/Z).

## Declaration of interest

A.J.P is Chair of UK Dept. Health and Social Care’s (DHSC) Joint Committee on Vaccination & Immunisation (JCVI), and was a member of the WHO’s SAGE until 2022. The views expressed in this article do not necessarily represent the views of DHSC, JCVI, or WHO. The University of Oxford has entered into a partnership with AstraZeneca on coronavirus vaccine development. The authors have no other relevant affiliations or financial involvement with any organisation or entity with a financial interest in or financial conflict with the subject matter or materials discussed in the manuscript. This includes employment, consultancies, honoraria, stock ownership or options, expert testimony, grants or patents received or pending, or royalties.

## Declaration of Competing Interest

The authors declare the following financial interests/personal relationships which may be considered as potential competing interests: Sir Andrew J. Pollard reports a relationship with UK Department of Health and Social Care’s Joint Committee on Vaccination and Immunisation that includes: non-financial support. Sir Andrew J. Pollard reports a relationship with World Health Organization that includes: non-financial support.

## Data Availability

Data will be made available on request.
