# Supplementary material for: Immunisation with purified Coxiella burnetii phase I lipopolysaccharide confers partial protection in mice independently of co-administered adenovirus vectored vaccines
Source: Vaccine. 2023 May 5;41(19):3047–57. doi: 10.1016/j.vaccine.2023.04.012 (PMC10914673; doi:10.1016/j.vaccine.2023.04.012)
Supplement: Supplementary data 1 [file mmc1.docx]

**Supplementary information**

**Supplementary table 1 Vaccine groups in the first challenge experiment and the mean body mass change post-challenge in mice receiving different adenoviral vaccine construct formulations.** Mice were challenged with aerosolised *C. burnetii.* Significance was calculated with one-way ANOVA for DPI = 7 (*P* < 0.0001) and DPI = 8 (*P* < 0.0001) followed by Tukey’s multiple comparisons test. *P <* 0.05 (*), *P <* 0.01 (**), *P <* 0.001 (***), *P <* 0.0001 (****).

| Group | Mean body mass change (%) | 95% CI | Significance of weight loss compared with naïve control (Adj. *P*) | |
| --- | --- | --- | --- | --- |
|  |  |  | DPI = D7 | DPI = D8 |
| Coxevac | 1.750 | 0.678-2.822 | <0.0001 (****) | <0.0001 (****) |
| Ad-GroEL+ Ad-CBU0091 + Ad-CBU1652 | 0.778 | -0.6691-2.225 | 0.3663 | 0.1905 |
| Ad-empty (7 weeks post immunisation) | -0.7338 | -2.313-0.8451 | 0.8042 | 0.9627 |
| Ad GroEL + Ad-P1 + Ad-YbgF + Ad-CBU0091 + Ad-CBU1652 | -0.7877 | -2.123-0.5473 | 0.9426 | 0.9066 |
| Ad-GroEL + Ad-P1 + Ad-YbgF + Ad-OmpH + Ad-SecB | -0.8554 | -2.237-0.5258 | 0.9971 | 0.7808 |
| Ad-GroEL + Ad-P1 + Ad-YbgF + Ad-CirD + Ad-CBU2007 | -1.032 | -2.874-0.8097 | 0.9996 | 0.9999 |
| Ad-GroEL + Ad-MIP + Ad-YbgF + Ad-OmpH + Ad-IcmK | -1.907 | -4.051-0.2370 | 0.8885 | 0.9862 |
| Ad-Com1 + Ad-GroEL + Ad-P1 + Ad-YbgF + Ad-CBU1157 | -2.353 | -4.036-0.6706 | >0.9999 | >0.9999 |
| Naïve | -1.208 | -3.177-0.7600 | NA | NA |
| Ad-GroEL + Ad-CBU0091 + Ad-CBU1652 (7 weeks post immunisation) | -0.6723 | -2.326-0.9817 | 0.9917 | 0.9627 |

**Supplementary table 2 Mean body mass change post-challenge in mice receiving different adenoviral vaccine constructs formualted with or without *C. burnetii* phase I LPS from the second challenge experiment.** Mice were challenged with an aerosol containing *C. burnetii.* Significance was calculated with one-way ANOVA for DPI = 7 (*P* < 0.0005) and DPI = 8 (*P* < 0.0001) followed by Tukey’s multiple comparisons test. *P <* 0.05 (*), *P <* 0.01 (**), *P <* 0.001 (***), *P <* 0.0001 (****).

| Group | Mean body mass change (%) | 95% CI | Significance of weight loss compared with Ad-Empty control (Adj. *P*) | |
| --- | --- | --- | --- | --- |
|  |  |  | DPI = 7 | DPI = 8 |
| Coxevac | -3.996 | -5.629 - -2.363 | 0.0038 (**) | 0.0002 (***) |
| Ad-01G fusion + LPS | -4.483 | -7.263 - -1.500 | 0.9515 | 0.2217 |
| LPS | -4.483 | -6.840 - -2.126 | 0.6426 | 0.0243 (*) |
| Ad-01 fusion + LPS | -4.636 | -7.285 - -1.986 | 0.7258 | 0.0418 (*) |
| Ad-01 fusion + Ad-GroEL + LPS | -5.100 | -8.012 - -2.188 | 0.9885 | 0.6567 |
| Ad-GroEL+ Ad-CBU0091 + Ad-CBU1652 + LPS | -5.788 | -9.231 - -2.346 | 0.9885 | 0.2603 |
| Ad-01 + GroEL | -6.405 | -7.263 - -1.500 | >0.9999 | 0.9851 |
| Ad-empty | -7.660 | -11.59 - -3.727 | NA | NA |
| Ad-GroEL + Ad-CBU0091 + Ad-CBU1652 | -7.716 | -11.67 - -3.765 | >0.9999 | 0.9997 |
| Ad-01G fusion | -8.876 | -12.97 - -4.786 | 0.9998 | >0.9999 |
